# Preserving Peri-Implant Soft Tissue Health: A Case Report on Immediate Implant Placement Using the Socket Shield Technique

**DOI:** 10.7759/cureus.57940

**Published:** 2024-04-09

**Authors:** Mohanasatheesh Shanmugam, Anitha Balaji, Mohan Valiathan, Rudhra Kannan, Angelin fiona Jeyaraj samuel

**Affiliations:** 1 Periodontics, Sree Balaji Dental College and Hospital, Chennai, IND

**Keywords:** peri-implant marginal tissue health, immediate implant, bundle bone, partial extraction therapy, socket-shield technique

## Abstract

Alveolar bone resorption is a natural occurrence following tooth extraction, complicating the process of prosthetic rehabilitation with implants. Techniques such as socket preservation, atraumatic extraction, and immediate implant placement are employed to reduce the dimensional changes associated with extraction. The socket shield technique (SST) is effective in preserving the alveolar ridge's contour, enhancing the aesthetic results of rehabilitation by maintaining the integrity of the bundle bone complex even when the buccal bone is less than 1mm.

This case report presents a 23-year-old female patient with a fractured upper central incisor. The socket shield technique was chosen based on the clinical findings from the cone beam computed tomography (CBCT) scan. Immediate temporization was provided to preserve soft tissue integration. A comparison of the initial and subsequent cone beam computed tomography (CBCT) scans, along with clinical observations, suggests that the socket shield technique is a viable method for preserving both hard and soft tissue structures in the anterior dental region, thereby improving aesthetic outcomes.

## Introduction

Tooth extraction causes dimensional changes in the alveolar ridge [1,2], which has an impact on prosthetic management and its emergence profile, especially in the esthetic zone due to the collapse of the “lamina dura-periodontal ligament” or “bundle bone” complex [3]. Within three months, nearly 40-60% of bone loss occurs, the most common of which involves the buccal bone plate [4]. To minimize this alveolar bone resorption and maintain the periodontal architecture, the placement of implants immediately after tooth extraction has been advocated [5]. Implantology is increasingly recognized as an effective treatment option for achieving biomimetic rehabilitation akin to natural teeth. However, placing implants in the anterior region remains a challenging endeavour [6]. 

Different techniques for preventing bone loss after extraction, such as an immediate implant, augmentation procedures [7], platform switching concepts, and various socket preservation techniques [8], such as “immediate dentoalveolar restoration” or the “ice cream cone” technique [9], have been investigated in the literature, but the peri-implant modifications still exist. Studies have shown that bundle bone resorption can be minimized if the dental root is preserved. The socket shield procedure introduced by Hürzeler et al. (2010) [10] is one of the techniques of partial extraction therapy for immediate implant placement. Preserving the coronal root portion coronal to the buccal bone plate prevents buccal bone resorption and achieves a stable restoration that fulfills the esthetic and functional needs of a patient. Osseointegration depends on integration of implant confinement into the bony walls, especially in contained defects, This environment can be achieved by using the socket shield technique. 

With the above ideas, this case report addressed the immediate implant placement using the socket shield technique in a young female patient to preserve the soft and hard tissues for enhanced esthetic tooth replacement.

## Case presentation

**Figure 1:**
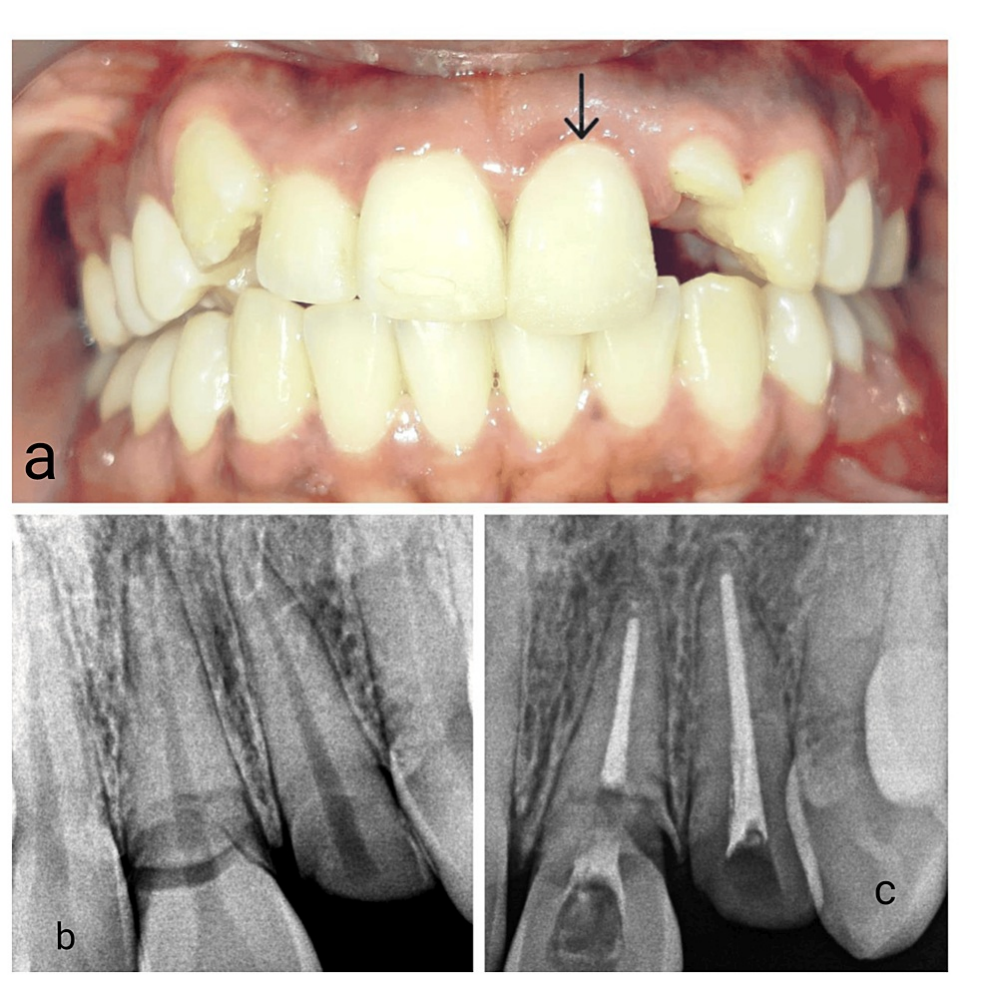
Preoperative clinical picture and X-ray (a) Preoperative clinical picture, (b) Preoperative digital X-ray showing fractured 21 at the cementoenamel junction, and (c) Digital X-ray showing endodontically treated 21.

A 23-year-old female patient presented with a chief complaint of broken upper front teeth 21 Federation Dentaire Internationale (FDI) notation (Figure 1A). The fracture was at the cementoenamel junction (Figure 1B). Soft tissue examination shows a low lip line and a medium gingival phenotype. A root canal treatment was performed to prevent potential future periapical pathology and to facilitate precise sectioning of the root (Figure 1C).

**Figure 2:**
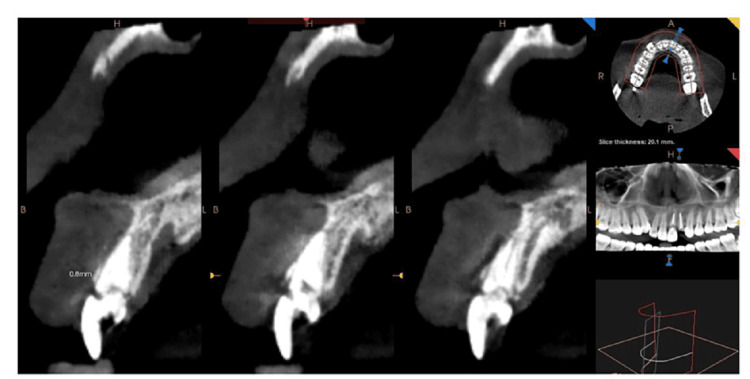
Preoperative CBCT scan image The preoperative cone beam computed tomography (CBCT) revealed a buccal bone thickness of less than 1 mm.

Following an initial assessment, the patient underwent a Cone Beam Computed Tomography (CBCT) scan (Figure 2). The scan showed a bone density of 744 Hounsfield Units (HU), 13.1 mm from the alveolar crest to the nasal floor, a 3.9 mm gap from the root tip to the nasal floor, and a buccal-lingual width of 6.2 mm. Based on these findings, a 4 mm wide and 14 mm long dentium implant was chosen to achieve primary stability. Due to the thin buccal bone plate observed in CBCT, the socket shield technique was selected for this patient to ensure optimal outcomes.

**Figure 3:**
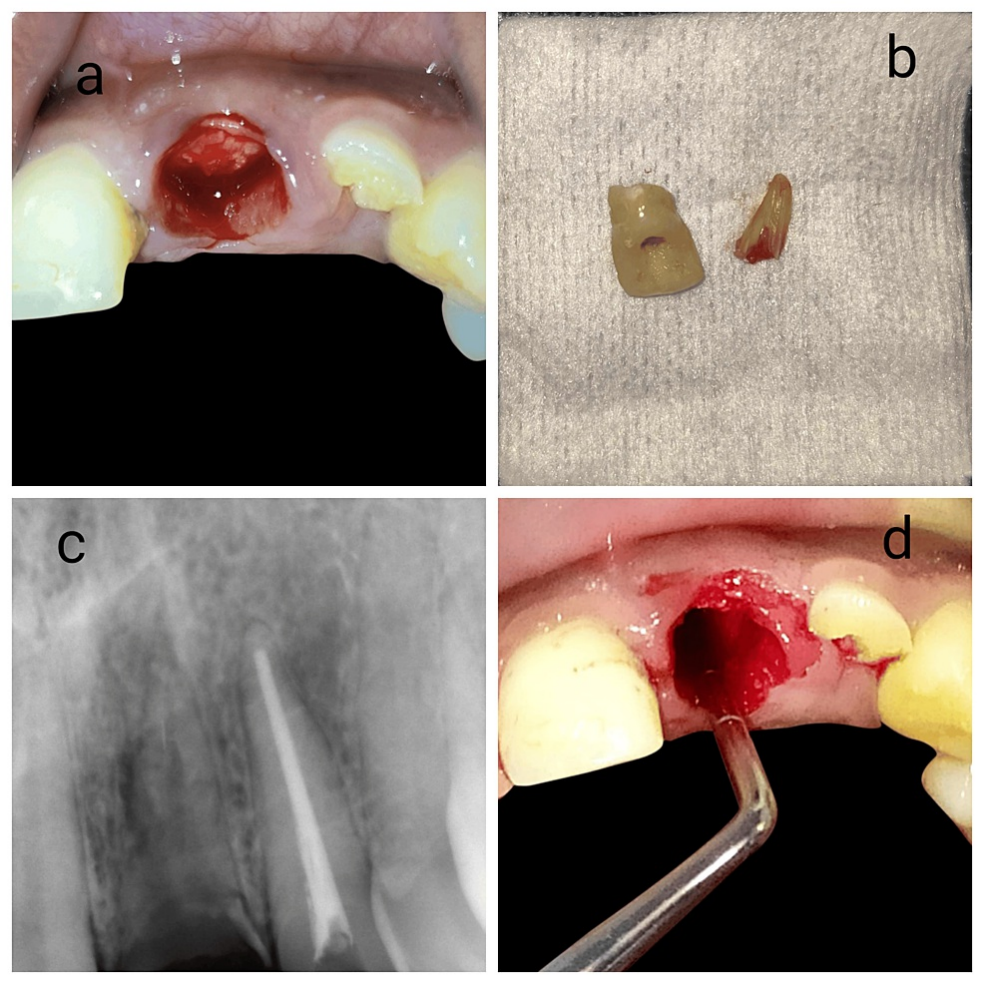
Extraction of palatal fragment retaining the buccal segment of the root and shield being created (a) Clinical picture taken after decoronation of the crown portion till to the crest of the margin gingiva, (b) Extracted 21 crown and a palatal portion of the root, (c) A digital X-ray was taken following the extraction of the palatal section of the root prior to preparation shield, (d) Clinical picture showing socket shield before reduction till to the level of the alveolar crest.

The patient received a comprehensive explanation of the procedure and consent was duly obtained. Prior to the procedure, standard preoperative measures were taken. The patient was administered 500 mg of amoxicillin and a 0.12% chlorhexidine rinse. Local anesthesia was given, and the fractured crown portion was removed. The root was then sectioned using a long shank Zekrya bur, splitting it mesiodistally in alignment with the tooth’s long axis. The periotome was used to sever the periodontal ligament (PDL) fibers to luxate the palatal fragments and was removed cautiously (Figures 3a-3d).

**Figure 4:**
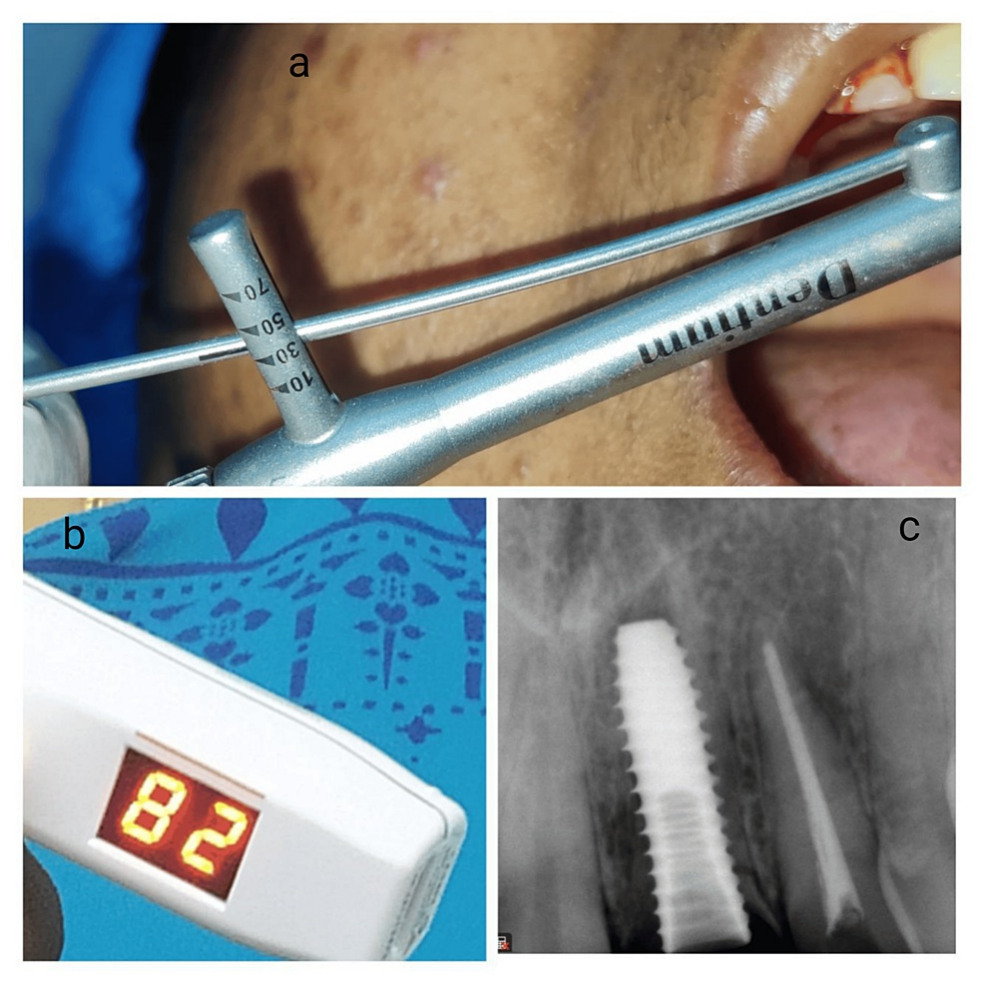
Implant placement with adequate primary stability and implant stability quotient value (a) Following the placement of the implant, an immediate torque of 45 Ncm was achieved, indicating adequate primary stability, (b) The Implant Stability Quotient (ISQ) value measuring 82 suggestive of good mechanical stability, (c) Immediate postoperative digital X-ray to check for Implant position.

It was ensured that the apical portion was removed to prepare a socket shield preventing contact from the implant shield was also reduced coronally and beveled till to the alveolar bone crest to make room for soft tissue growth. After removing the palatal portion, an osteotomy was performed using a 2 mm pilot drill, followed by subsequent drills to prepare for implant placement. The implant’s contact with the surrounding mesial, distal, and palatal bone walls was verified, and its stability was measured with an Implant Stability Quotient (ISQ) using an Osstell monitor, achieving an initial Implant stability quotient (ISQ) of 82 and a torque of 45 Ncm (Figures 4a-4c).

**Figure 5:**
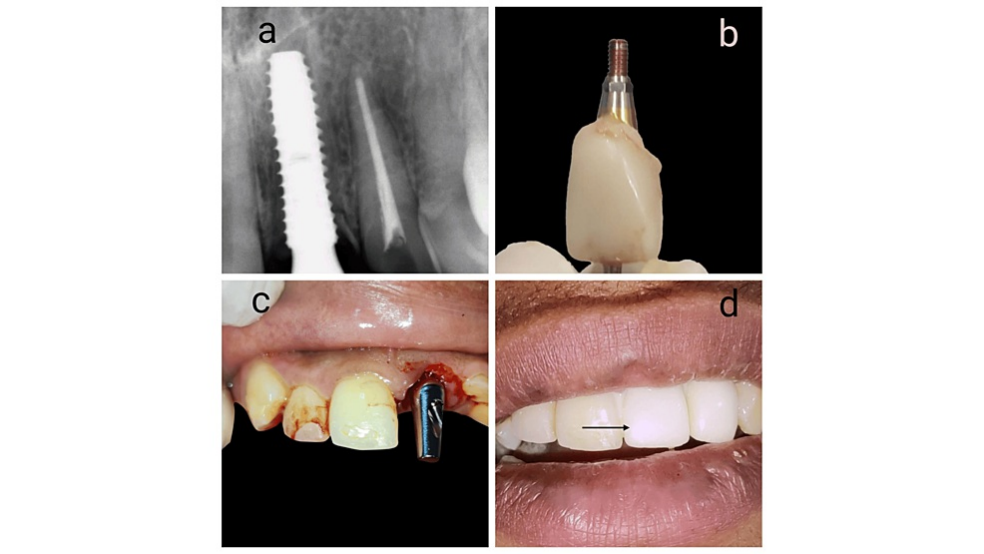
Immediate chair chide temporization (a) Digital X-ray taken following straight abutment placement, (b) A temporary restoration was crafted chairside to preserve the health of the peri-implant soft tissues, (c) Clinical picture showing the straight stock abutment in position, (d) A clinical photograph illustrates the temporization that was provided immediately after the implant placement.

The gap was filled with Beta tricalcium phosphate bone graft material and covered with a membrane. A straight abutment was then attached, and a screw-retained temporary crown was crafted chairside to help with soft tissue remodeling (Figures 5a-5d). The occlusion was adjusted to ensure non-functional loading. Postoperative care instructions included the continuation of antibiotics, NSAIDs, and chlorhexidine mouthwash.

**Figure 6:**
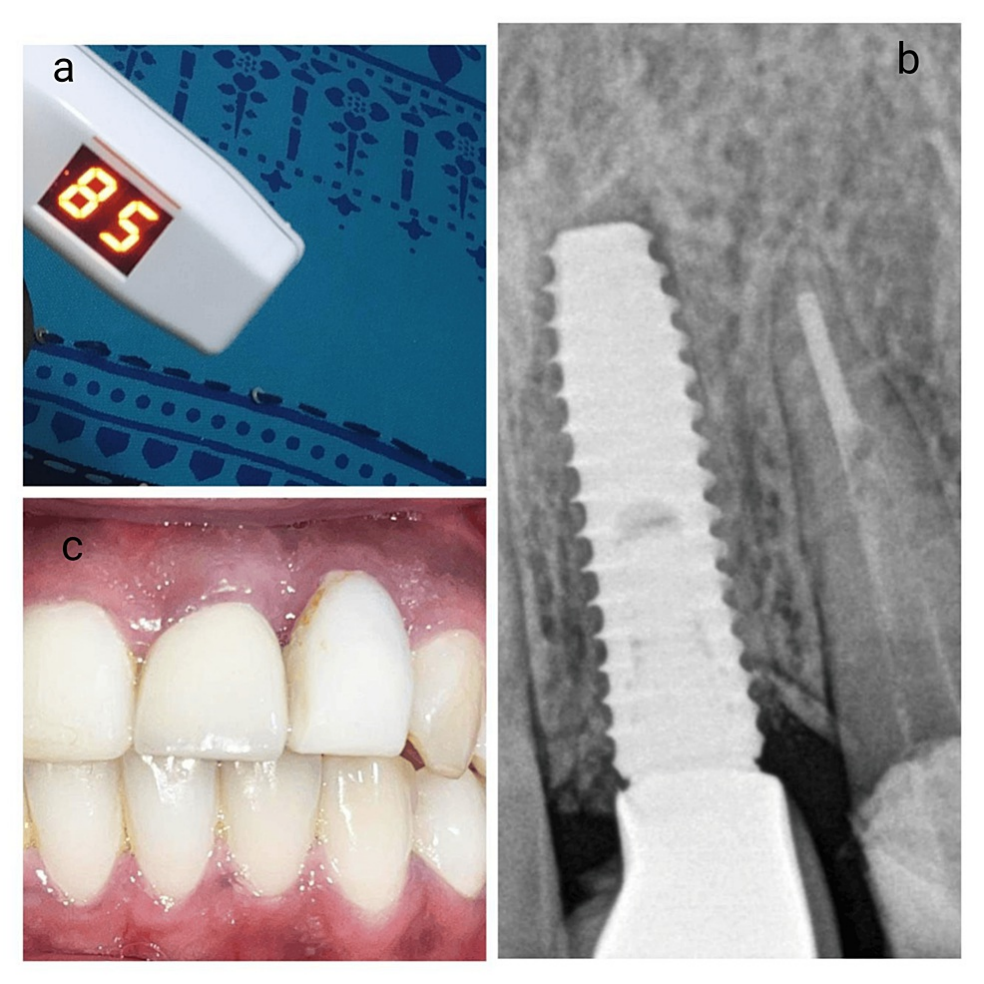
Three months clinical and post operative X-ray of final prosthesis in relation to 21 with implant stability quotient value (a) Three months after the implant placement, the Implant Stability Quotient (ISQ)  measured was 85. This value indicates a high level of biological stability for the dental implant, (b) Digital X-ray showing the implant with the final prosthetic crown in relation to 21, (c) A clinical picture of 21 with porcelain fused metal crown.

Three months post-procedure, the patient’s condition was satisfactory. At this follow-up, the temporary crown was replaced with a screw-retained, porcelain-fused-to-metal crown (Figures 6a-6d).

**Figure 7:**
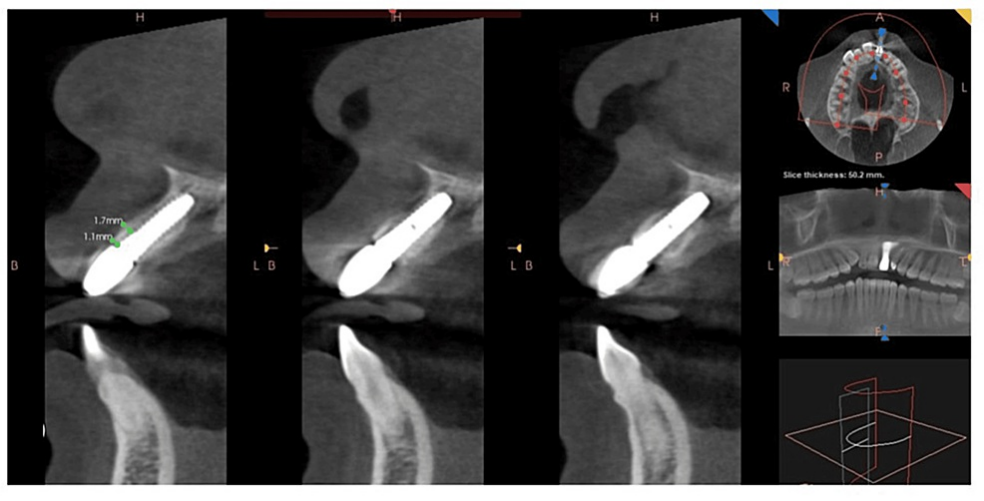
Postoperative CBCT pictures The six-month post-cone beam computed tomography (CBCT) shows the intact presence of a shield with adequate hard and soft tissues around the implant. .

The cone beam computed tomography (CBCT) scan taken six months later revealed the proper sagittal position of the implant and the presence of the facial shield and showed an improved bone density of 1173 HU, a measurement from the alveolar crest to the nasal floor of 12.7 mm, and from the root apex to the nasal floor of 2.2 mm. The buccal-lingual width was recorded at 6.4 mm (Figure 7).

**Figure 8:**
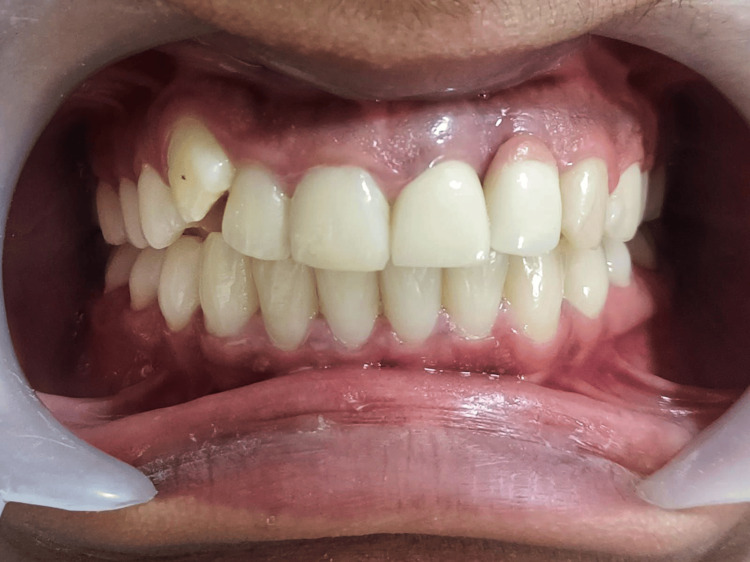
One-year follow-up clinical picture The clinical observation indicates a well-defined stippling and mucosal zenith around tooth number 21, demonstrating the stable health condition of the peri-implant soft tissues.

The patient was then recalled after a year for a routine follow-up thereafter (Figure 8).

## Discussion

Hurzeler et al., for the first time, reported the socket-shield technique as a proof of concept in an animal model [10]. While they demonstrate the formation of a bony layer between the socket shield and the implant surface through histological evaluation, the animal model poses limitations when the technique is translated to humans. Our case report demonstrated that Hurzeler et al.'s hypothesis of retaining root fragments does not interfere with osseointegration in implant placement in extraction sockets. Van der Weijden et al. observed the dimensional changes in alveolar height and width after tooth extraction; the reduction in width of the alveolar ridges was 3.87 mm [11]. The mean clinical mid-buccal height loss was 1.67 mm.

In the maxillary aesthetic zone, the buccal bone, comprising both bundle and cortical bone, typically measures less than 1 mm in thickness. The bundle bone, which receives most of its blood supply from the periodontal ligament (PDL) vasculature, is prone to an average loss of 0.3-0.5 mm. Tooth extraction in this area often results in the loss of facial bone, particularly in the crestal region, more so than in the palatal contour, due to the depletion of bundle bone. To mitigate this, preserving a fragment of the buccal root and shaping it into a shield can maintain the bundle bone’s blood supply, a strategy known as the biological cheating phenomenon. The findings of our case report strongly corroborate the aforementioned rationale.

A study conducted by Bramanti et al. (2018) using SST for a single implant showed better marginal bone levels and Pink Esthetic Score (PES) values after a three-year follow-up [12]. Oliva et al. (2023), in a systematic review, analysed the mean values of marginal bone loss (0.39±0.28 mm), pink esthetic score (12.08±1.18), buccal bone plate resorption (0.32±0.10 mm) and implant survival rate of 98.6% [13]. 

Gluckman et al., 2017, in a retrospective study with 128 patients with SST conducted for a year, analysed biological complications of 16 exposures, three infections, and one migration (19.5%) with a 96.1% survival rate [14]. Glocker et al.'s study in 2014 demonstrated a modified socket shield technique where implant placement was deferred for six months, resulting in the successful preservation of the buccal lamellar bone in all cases [15]. This approach offers a practical alternative for situations where immediate implantation is not viable. 

The “root membrane technique” by Sirompas et al. is a dental procedure that retains part of the buccal root to prevent bone loss and involves the implant making contact with this root fragment [16]. Their study reported successful implant integration in all 46 patients over a five-year period, with only one instance of root resorption that did not affect the implant’s performance. Sun et al. (2020), in a randomized clinical study, assessed no complications and a 100% survival rate with 30 implants for a three-year period [17]. Similarly, Tiwari et al. observed 100% survival for one year with a mean marginal bone loss of socket shield technique (SST): 0.03±0.02 [18]. 

According to a review by Sharma et al., the implant survival rate may be comparable in both the socket shield technique and the conventional technique, but the socket shield technique seems to perform better in terms of bone preservation, esthetic outcome, and patient satisfaction [19]. Gharpure et al.'s systematic review analyzed the biological plausibility and long-term clinical prognosis. Complications like pocket formation, inflammation, mucositis, and peri-implantitis buccal/crestal bone loss (78.78%) and shield exposure/failure (12.12%) were reported [20]. 

Our case report demonstrated a well-defined mucosal zenith in relation to tooth 21 and stippling, indicative of good peri-implant health free of inflammation. The six-month postoperative cone beam computed tomography scan revealed minimal marginal bone loss. Additionally, a comparison between the preoperative and postoperative scans and clinical parameters demonstrated both the preservation of soft and hard tissues around the implant.

## Conclusions

This case report of immediate implant placement using the socket shield technique demonstrates how an immediate implant can be successfully restored by maintaining both the peri-implant hard and soft tissue architecture. The biological concept of the socket shield may appear to be efficient in preserving the peri-implant hard tissue dimensions, but long-term results remain scarce, and well-designed clinical studies with long-term follow-up are required before recommendation for routine clinical practice, in addition to the requirement of highly technique-sensitive skill by the clinician. 
